# The role of bronchoalveolar lavage fluid metagenomic next-generation sequencing in detecting pathogens and optimising antibiotic therapy in paediatric severe community-acquired pneumonia

**DOI:** 10.3389/fcimb.2025.1688473

**Published:** 2026-01-14

**Authors:** Yali Xu, Yanyan Ma, Qian Huang, Xixia Guo, Luyang Guo, Yishuai Ren, Weihong Lu, Xiangtao Wu, Duoduo Li, Shujun Li

**Affiliations:** Department of Pediatrics, The First Affiliated Hospital of Xinxiang Medical University, Xinxiang, Henan, China

**Keywords:** bronchoalveolar lavage fluid, detecting pathogens, paediatric, pneumonia, prognosis

## Abstract

**Background:**

Severe community-acquired pneumonia (SCAP) remains a major cause of mortality in the paediatric population, with current diagnostic and treatment approaches often proving insufficient and contributing to the growing challenge of antibiotic resistance. This study explored the potential of metagenomic next-generation sequencing (mNGS) of bronchoalveolar lavage fluid as a tool to enhance the precision of antibiotic management in children with SCAP.

**Methods:**

A retrospective cohort study of 202 paediatric patients with community-acquired pneumonia (aged 1 month–18 years) admitted to the First Affiliated Hospital of Xinxiang Medical University (November 2020–March 2023) was conducted. Patients were grouped by severity (intensive care unit [ICU]/non-ICU) and mNGS timing (early: ≤72 hours post-admission; late: >72 hours). The diagnostic efficacy of mNGS versus conventional microbiological techniques (CMT) was evaluated using sensitivity, specificity, positive/negative predictive values and area under the receiver operating characteristic curve (AUC) analysis. Antibiotic adjustments and clinical outcomes were analysed via survival statistics.

**Results:**

Metagenomic next-generation sequencing showed a higher positive detection rate (98.51%) than CMT (47.52%) (AUC = 0.82, 95%CI: 0.76–0.88). Of the 202 patients, 127 (62.87%) were male, with a median age of 1.88 years (interquartile range: 0.29–7 years). Early mNGS was associated with fewer extrapulmonary complications (69.63% vs 55.22% in the late group, *p* < 0.05), and shorter hospitalisation (median 13 vs 15 days, *p <*0.01). Antibiotic escalation occurred in 50 (24.75%) cases, de-escalation in 22 (10.89%) and same-level adjustment in 25 (12.38%).

**Conclusion:**

Metagenomic next-generation sequencing outperforms CMT in pathogen detection. Early mNGS is associated with improved clinical outcomes, suggesting its potential utility in paediatric SCAP management.

## Introduction

Severe community-acquired pneumonia (SCAP) is a significant cause of morbidity and mortality in children aged <5 years (Bradley et al., 2011), particularly in developing countries where mortality rates can reach 20%–50% (Smith et al., 2021; Chen S. et al., 2022). Globally, pneumonia accounted for 22% of deaths in children aged 1–5 years in 2019 (Collaborators G L, 2022), underscoring its critical impact on child health. The challenge of timely and accurate pathogen identification, coupled with rising antibiotic resistance, highlights the need for improved diagnostic strategies to optimise treatment and reduce mortality (Larsson and Flach, 2022; Lin et al., 2023).

Current diagnostic approaches rely heavily on conventional microbiological techniques (CMT), such as bacterial culture and polymer chain reaction (PCR), which have significant limitations. Bacterial culture requires 48–72 hours for results, which delays targeted therapy (Lin et al., 2023), and PCR is restricted to preselected pathogens, missing those that are rare or unexpected (Qu et al., 2022). Both methods frequently fail to detect mixed infections (e.g. bacterial–viral co-infections), which are common in paediatric SCAP and linked to poorer outcomes (Zheng et al., 2021). These gaps lead to empirical overuse of broad-spectrum antibiotics, with 30%–40% of paediatric community-acquired pneumonia (CAP) cases now resistant to first-line agents such as ampicillin and ceftriaxone (Wei et al., 2023).

Metagenomic next-generation sequencing (mNGS) of bronchoalveolar lavage fluid (BALF) is a promising tool that offers several advantages over traditional methods, including higher sensitivity and specificity, shorter turnaround time, wider pathogen coverage and the potential for antibiotic resistance detection (Chen S. et al., 2022; Yang et al., 2022; Liang et al., 2024). Furthermore, BALF mNGS can provide valuable insights into the microbial community in the lower respiratory tract, which may impact the pathogenesis and prognosis of CAP (Thibeault et al., 2021). However, critical knowledge gaps remain; its optimal timing in paediatric SCAP, impact on antibiotic stewardship (e.g. de-escalation vs escalation) and correlation with long-term outcomes (e.g. recurrence) are underexplored. Most existing studies focus on adult populations or general paediatric infections, leaving the specific utility of mNGS in paediatric SCAP poorly defined (Chen et al., 2024; Su et al., 2024).

This study aims to address three key questions: (1) How does the diagnostic performance of BALF mNGS compare with CMT in detecting pathogens in paediatric SCAP, specifically in terms of sensitivity, specificity and coverage of mixed infections? (2) What is the impact of early mNGS (within 72 hours of admission) versus late mNGS (after 72 hours) on clinical outcomes, including antibiotic adjustment rates, extrapulmonary complications and hospital length of stay (LOS)? (3) Can mNGS-guided antibiotic adjustments reduce unnecessary broad-spectrum antibiotic use? We hypothesise that mNGS can outperform CMT, early testing improves outcomes and guided adjustments enhance treatment precision. The findings aim to inform clinical practice by validating mNGS as a tool to optimise paediatric SCAP management and mitigate antibiotic resistance.

## Research methods

### Participants

The sample size was determined based on the primary outcome of pathogen detection rate, with reference to previous studies on mNGS in respiratory infections (Tao et al., 2022; Wei et al., 2023). Assuming a two-tailed *α* of 0.05, power of 80% and an expected difference in positive detection rates between mNGS and CMT of 30%, the minimum required sample size was calculated as 186 cases. At least 200 cases should be included to address the possible cases of loss to follow-up or incomplete data.

A retrospective cohort analysis was conducted on patients with CAP who were admitted to the Paediatrics Department of the First Affiliated Hospital of Xinxiang Medical University between November 2020 and March 2023 and underwent BALF mNGS. The inclusion criteria were as follows:

(1) Age ranging from 1 month to 18 years; (2) meeting the diagnosis of CAP (Nascimento-Carvalho, 2020); (3) fulfilling the diagnostic criteria for SCAP (Bradley et al., 2011). The presence of any one of the following items is considered as SCAP: poor general condition, food refusal or dehydration, disturbance of consciousness, significantly increased respiratory rate (>70 times/minute for infants or >50 times/minute for older children), central cyanosis, dyspnoea (groaning, nasal flaring, three-concave sign), multiple lobes or ≥2/3 of unilateral lung involvement, pleural effusion, pulse oximetry ≤ 0.92 (at sea level) or extrapulmonary complications.

Indications of bronchoalveolar lavage (BAL) include the following (Alpert and Htwe, 2024): (1) recurrent or persistent wheezing; (2) localised stridor; (3) unexplained chronic cough; (4) recurrent respiratory tract infections; (5) haemoptysis; (6) difficulty weaning from the ventilator; (7) chest imaging abnormalities, such as tracheal and bronchopulmonary dysplasia and/or malformation, pulmonary consolidation and atelectasis, emphysema, pulmonary mass lesions, diffuse pulmonary disease, pneumomediastinum, airway and mediastinal space occupation and pleural cavity lesions requiring differential diagnosis; (8) aetiological diagnosis and treatment of pulmonary infectious diseases.

The exclusion criteria for this study were as follows: (1) cases where the attending physician suspects that the cause of the condition is due to other pathogens or foreign bodies; (2) patients with chronic diseases, such as bronchiectasis, asthma or malignant tumours, or those admitted to the paediatric intensive care unit (ICU) due to cardiac arrest, organ transplantation or surgery; patients with hospital-acquired infections, immune deficiency defined as HIV infection, primary immunodeficiency or other conditions that lead to a compromised immune system or those using immunosuppressive drugs are also excluded; (3) individuals who are intolerant to BAL; (4) cases with incomplete information.

### Data collection

All 202 patients enrolled in this study met the predefined diagnostic criteria for SCAP. Within this SCAP cohort, patients were further categorised into an ICU group and a non-ICU group based on clinical decisions regarding the level of care required during hospitalisation. Admission to the ICU typically reflected more critical illness, including the need for mechanical ventilation, vasopressor support or intensive monitoring, thereby serving as a proxy for higher disease severity within the SCAP spectrum. This subgroup stratification was performed to evaluate differences in pathogen profiles, treatment responses and outcomes across levels of care intensity.

Alternatively, patients were categorised into early mNGS group (mNGS was performed ≤72 hours of admission) and late mNGS group (mNGS was performed >72 hours after admission) based on the timing of mNGS detecting. During the study period, a total of 214 extraction/library negative controls were processed alongside patient samples. Among these, 208 showed no detectable microbial reads above the threshold, and the remaining 6 had low-level detections of known contaminants (e.g. *Cutibacterium acnes*, *Corynebacterium striatum*), none of which were reported as clinically relevant pathogens.

The data collection was comprehensive, encompassing a wide array of variables. These included demographic details and clinical manifestations, such as fever, cough and wheezing. Laboratory test results were also collected, which included pathogen identification, white blood cell count, inflammatory markers, lactate dehydrogenase (LDH), alanine aminotransferase (ALT) and urea nitrogen levels. In addition, the severity of illness was evaluated using the paediatric-specific versions of established scoring systems: the paediatric versions of the Acute Physiology and Chronic Health Evaluation (APACHE II) (Chhangani et al., 2015) and the Sequential Organ Failure Assessment (SOFA) scores (Matics and Sanchez-Pinto, 2017). Information was also gathered on the application and adjustment of antibiotics before and after mNGS. The data collection further incorporated bacterial culture results along with corresponding drug sensitivity reports. The mNGS results and associated drug resistance gene reports were also included. Finally, the length of hospital stay for each patient was documented.

### Antibiotic adjustment criteria

Antibiotic adjustments were classified as escalation, de-escalation or same-level based on the spectrum of activity and clinical guidelines. Escalation was defined as switching from narrow-spectrum to broad-spectrum antibiotics or from lower to higher antibiotic classes (e.g. penicillin to carbapenem). De-escalation involved transitioning from broad-spectrum to narrow-spectrum antibiotics or from higher to lower classes (e.g. carbapenem to penicillin). Same-level adjustments referred to changes within the same antibiotic class or spectrum (e.g. ceftriaxone to cefotaxime). These classifications were determined based on pathogen identification, antimicrobial susceptibility testing and clinical judgment.

Antibiotic therapy adjustments were categorised into three types based on the primary rationale derived from mNGS and complementary testing:

Pathogen-directed de-escalation – initiated when mNGS identifies a specific pathogen with predictable antimicrobial susceptibility, allowing narrowing of broad-spectrum coverage. For example, detection of *Mycoplasma pneumoniae* led to discontinuation of β-lactams and initiation of macrolides; identification of influenza virus prompted cessation of antibiotics and initiation of oseltamivir.

Resistance gene–driven escalation or modification – performed when mNGS detects known resistance genes, even in the absence of culture confirmation. Examples include the following:

Detection of the mecA gene → switch from a methicillin-sensitive regimen to vancomycin or linezolid.

Presence of the ermB or msrA genes in *Streptococcus pneumoniae* → avoid macrolides or consider alternative agents.

Identification of extended-spectrum β-lactamase (ESBL) genes (blaCTX-M, blaTEM) → escalate to carbapenems or β-lactam/β-lactamase inhibitors.

Empirical modification guided by mNGS findings – applied when mNGS suggests a likely pathogen not initially covered and clinical course supported a change (e.g. adding antifungal agents upon detection of *Aspergillus fumigatus* in a persistently febrile, immunocompromised child, despite negative culture).

All adjustments were made by the attending physician in consultation with infectious disease specialists, integrating mNGS data with clinical status, inflammatory markers and available culture-based drug susceptibility results. This classification system was prospectively recorded and used for subsequent analysis.

This comprehensive data collection aims to provide a robust foundation for the analysis.

### Bronchoscopy procedure

Bronchoscopy was performed using flexible fibreoptic bronchoscopes with an outer diameter of 2.8–3.5 mm (Olympus BF-P180) (Olympus America Inc., Center Valley, PA, USA), selected based on patient age and airway size. All procedures were conducted under monitored anaesthesia care or general anaesthesia, with an endotracheal intubation or laryngeal mask airway used as needed to ensure airway patency and physiological stability, particularly in infants aged <1 year.

Bronchoalveolar lavage was performed in the involved lung segment under direct visualisation. Sterile saline, pre-warmed to 37°C, was instilled in 3–5 aliquots of 1 mL/kg per increment, with each aliquot ≤3 mL to prevent overdistension in young infants. For example, in a 4-kg infant, each flush was limited to 3–4 mL (≤3 mL per aliquot), administered slowly with immediate gentle aspiration following each instillation.

The negative pressure for aspiration was maintained between 6.65–13.3 kPa (50–100 mmHg), and the total recovered volume was recorded. A recovery rate of ≥40% was considered adequate for analysis (Schramm et al., 2021). The collected BALF was immediately placed on ice and stored at −20°C until further processing.

This protocol adheres to established paediatric bronchoscopy guidelines from the American Thoracic Society and European Respiratory Society, which support the safe performance of BAL in infants and young children when performed by experienced operators using appropriate equipment and physiological monitoring. Special attention was paid to minimising airway trauma, maintaining oxygenation and avoiding fluid overload in this vulnerable population (Faro et al., 2015).

### Conventional microbiological techniques

The culture and identification of BALF were performed using the BACTEC-FX automatic blood culture instrument and PhoenixTM100 automatic microbial analysis system (Becton, Dickinson and Company, USA), as per the manufacturer’s instructions. The susceptibility of antimicrobial drugs was assessed using the Kirby–Bauer method, in line with the Clinical Laboratory Standards Institute guidelines (CLSL, 2022). Polymer chain reaction analysis, conducted using the Roche LightCycler 480II (USA), facilitated the detection of DNA/RNA from respiratory syncytial virus (RSV), influenza A and B viruses and *M. pneumoniae*. This comprehensive approach ensures a robust and accurate analysis of the pathogenic landscape in patients with SCAP.

### Metagenomic next-generation sequencing

The mNGS method used for diagnosing pneumonia was implemented according to standardised operating procedures with the following detailed steps: (1) sample processing – a 1 mL sample of BALF was centrifuged at 12,000 × g for 5 minutes to collect microbial and human cells; (2) DNA was extracted using the TIANamp Micro DNA Kit (TIANGEN DP316) following the kit’s instructions, including steps of cell lysis, protein precipitation and DNA elution; (3) library construction – a DNA library was constructed using the NGS Library Construction Kit (Enzymatics) with unique dual index adapters. The process included end repair, A-tailing, adapter ligation and PCR amplification; (4) library purification – the library was purified using AMPure XP beads (Beckman Coulter) at 0.6x concentration and eluted in 20 µL of 1M Tris-HCl buffer; (5) sequencing – the purified library was then sequenced on the Illumina NextSeq 550 sequencing platform, using a single-end 75 bp sequencing strategy, with a target sequencing depth of 5 million reads per sample.

### Data analysis of metagenomic next-generation sequencing

The Bcl2 fastq software package was utilised to convert the base call sequence data of each sample into a fastq format file. Subsequently, the original reads were filtered using fastp and kz software to eliminate reads containing adapter contamination, low-quality and low-complexity reads. The filtered reads were then aligned to a human-related reference genome database, which included hg38 and non-human primer genome sequences, with human genome sequences being removed. The remaining reads were compared against non-redundant databases of bacteria, viruses, fungi and parasites using the Scalable Nucleotide Alignment Program. Taxonomic information for the species was generated by annotating the reads on the alignment and counting the number of species-specific sequences using an in-house program. The taxonomic annotation process involved two steps: first, comparing the reads with a pathogenic microorganism genome database using snap software, and second, performing final taxonomic annotation on the reads. The lowest common ancestor algorithm was employed when a sequence read could be compared with multiple reference genomes of different species simultaneously to classify specific reads. Additionally, samtools and bedtools were used to calculate the coverage and average sequencing depth of the species’ genome, serving as reference indicators for the reliability of the tested species. If there were fewer than three detected reads for a species, NT alignment was used to further validate the detection results (Shi CL. et al., 2020).

Genome-wide data on pathogenic microorganisms were collected from public databases. All species in the pathogen reference database were sourced from reputable publications, such as ‘Handbook of Clinical Microbiology’, ‘Clinical Microbiology Diagnosis and Instructions’ and the National Center for Biotechnology Information (NCBI) RefSeq genome database. Custom criteria, developed with reference to NCBI genome exclusion rules, were applied to remove low-quality genomes. Only high-quality representative strains downloaded from the NCBI RefSeq genome database or NCBI GenBank genome database were selected (Shi T. et al., 2020; Hu et al., 2023).

### Criteria for the determination of the metagenomic next-generation sequencing results

To ensure accurate detection, a systematic approach was followed when filtering the initially detected bacteria. First, any obvious sequence alignment anomalies and background contamination bacteria were eliminated. Next, the pathogen data were analysed and positive determinations were made. The obtained microbial list was compared with an internal background database, which includes microbes that have been observed in >50% of samples in the laboratory over the past 3 months. However, microbes detected in the negative control group or present in ≥25% of samples in the past 30 days were excluded if the detected species-specific reading number (SSRN) was ≥10 times that of the negative control group or other microbes. Additionally, microbes present in ≥75% of samples in the past 30 days were excluded. By removing suspicious background microbes from the microbial list, the accuracy of the results is improved. For different types of microbes, specific thresholds were set. Extracellular bacteria/fungi were required to rank in the top 10 among bacteria, fungi or parasites. Intracellular bacteria (excluding *Mycobacterium tuberculosis* and *Brucella*) and *Cryptococcus* were required to have an SSRN ≥ 10 (reads per million [RPM] ≥ 0.5) and rank in the top 10 among bacteria or fungi. The criteria applied were as follows: SSRN ≥ 10 for intracellular or fastidious bacteria (e.g. *M. pneumoniae, Chlamydia pneumoniae*), SSRN ≥ 3 for viruses and RPM ≥ 0.5, with additional consideration of taxonomic abundance and mapping quality. Pathogens detected in the negative control group or present in ≥25% of samples in the past 30 days were excluded if the detected SSRN was ≥10 times that of the negative control group or other microbes. These thresholds are crucial in accurately identifying and excluding background microbes, thereby improving the overall accuracy of the detection process (Shi T. et al., 2020).

### Long-term follow-up protocol

To evaluate the long-term health outcomes and recurrence of pneumonia, all patients were followed up for 6 months after discharge. Follow-up data were collected through outpatient visits at 1 month, 3 months and 6 months post-discharge, supplemented by telephone interviews if in-person visits were unavailable. The following long-term outcomes were recorded: (1) recurrence of pneumonia – defined as re-admission with clinical and radiological evidence of pneumonia within 6 months of discharge (Hu et al., 2023); (2) long-term sequelae – including chronic cough, wheezing or abnormal pulmonary function (assessed via pulse oximetry and, in children aged ≥6 years, spirometry); (3) antibiotic use after discharge – duration and type of antibiotics prescribed for post-discharge prophylaxis or treatment.

### Statistical analysis

Statistical analyses were conducted using SPSS 26.0 software. Non-normally distributed variables were expressed as median values with interquartile ranges, and intergroup differences were assessed using the Mann–Whitney U test. Categorical variables were presented as percentages, with Pearson’s chi-squared test or Fisher’s exact test used to evaluate differences between groups. The predictive values of mNGS, both positive and negative, were calculated relative to CMT. Pearson correlation analysis was utilised to investigate the relationship between the timing of mNGS and the adjustment of antibiotic treatment and Spearman correlation analysis was additionally performed for variables that did not satisfy the assumptions of linearity. Survival curves were generated using R 4.2.2, and Kaplan–Meier analyses were used to evaluate the influence of mNGS on survival and the length of hospital stay.

Multivariate regression analysis was performed to adjust for potential confounding factors, including the following: (1) demographic factors – age, gender; (2) disease severity indicators – SOFA score, APACHE II score; (3) inflammatory markers – C-reactive protein (CRP), procalcitonin (PCT), interleukin-6 (IL-6); (4) comorbidities – myocardial damage, anaemia, liver damage; (5) treatment-related factors – use of invasive/non-invasive ventilators, initial antibiotic types. Sensitivity analyses were conducted by excluding patients with extreme values or incomplete data to verify the stability of the results.

The effect of early mNGS on extrapulmonary complications and antibiotic adjustment was analysed using Cox regression, with adjustments made for potential confounding factors, such as age and underlying diseases. A *p*-value of <0.05 was considered statistically significant.

Furthermore, Kaplan–Meier survival curves were used to compare the time to first pneumonia recurrence between the early and late mNGS groups. Cox proportional hazards regression was performed to adjust for confounding factors (age, disease severity and antibiotic adjustment during hospitalisation) when analysing the impact of mNGS timing on long-term outcomes. A *p*-value of <0.05 was considered statistically significant.

## Results

### Characteristics of basic information on samples and patient conditions

**Figure 1:**
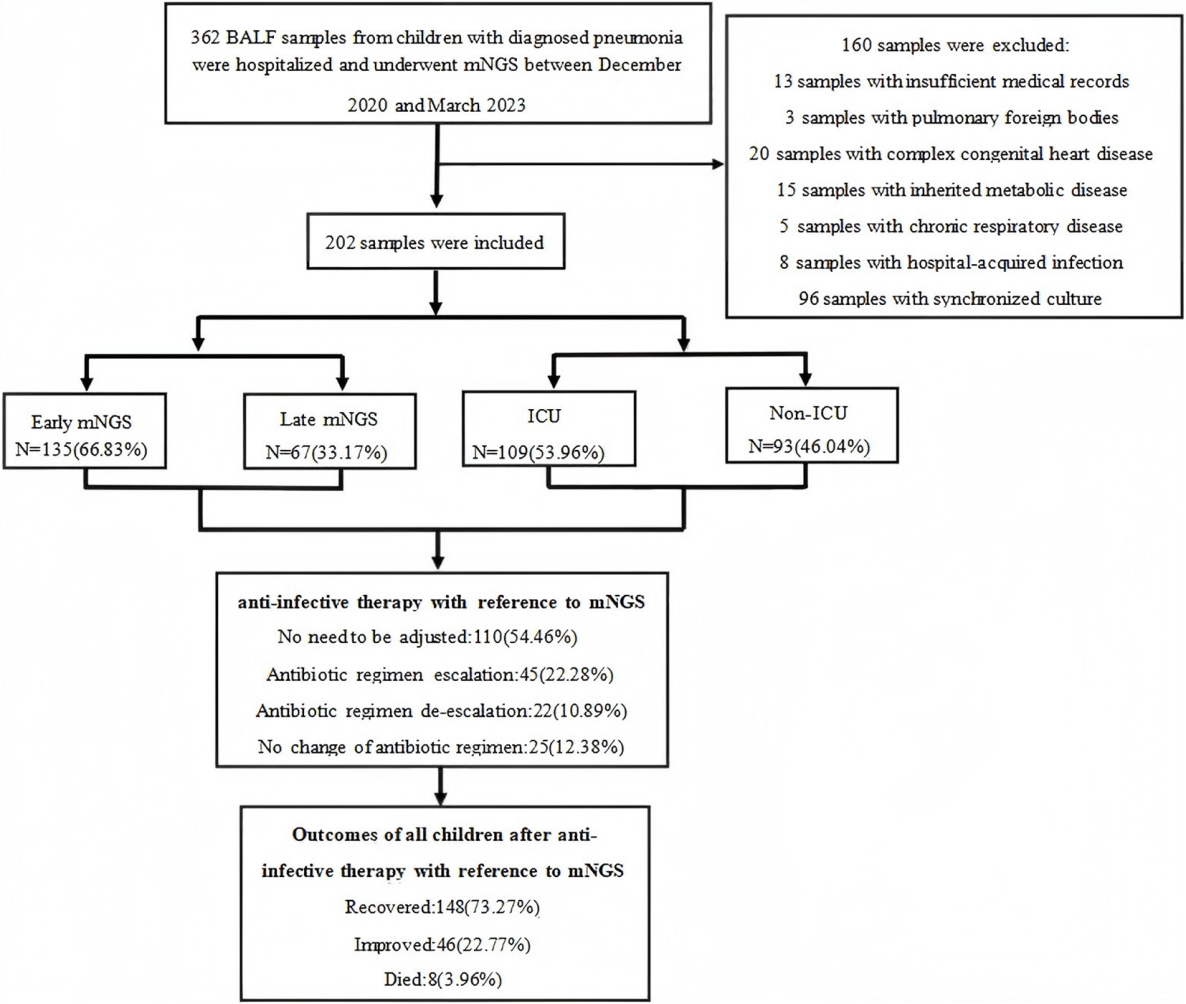
Research flow chart.

The exceptionally high mNGS positivity rate in this cohort reflects not only the invasive nature of BALF sampling but also the robust bioinformatic and laboratory protocols designed to suppress false positives while maximising true signal detection. The BALF mNGS testing was conducted on 362 pneumonia cases. Initial screening involved all 362 cases. After the exclusion of 160 cases due to non-infectious causes/chronic underlying diseases, a total of 202 cases were finally included. Among the included cases, 109 (53.96%) were from the ICU group and 93 (46.04%) were from the non-ICU group. The sample population consisted of 127 boys (62.87%) and 75 girls (37.13%). The median age was 1.88 years, with 12 patients (5.94%) at 1 month, 65 patients (32.18%) between 2 and 11 months, 64 patients (31.68%) aged 6–18 years and 61 patients (30.20%) aged 1–5 years. Clinically, the findings were consistent with clinical practice in 198 cases (98.02%) and not consistent in 4 cases (1.98%). In the ICU group, 39 patients (19.31%) required invasive ventilators, and 65 patients (32.18%) used non-invasive ventilators. Overall, 135 patients (66.83%) underwent early mNGS testing, and 67 patients (33.17%) underwent late mNGS testing. After the mNGS testing, adjustments in antibiotics were required for 92 cases (45.54%) but not for 110 cases (54.46%). Among these cases, 86 (42.57%) used restricted antibiotics, and 116 (57.43%) used unrestricted antibiotics. In terms of outcomes, 131 cases (64.85%) had extrapulmonary complications, and 71 cases (35.15%) had no complications. Additionally, 148 cases (73.27%) were cured, 46 cases (22.77%) showed improvement and 8 cases (3.96%) resulted in death (**Figure 1**, **Table 1**).

**Table 1:** Clinical data of included cases.

Index	Clinical value
Male, n(%)	129 (63.86)
Female, n(%)	75 (37.13)
Age, median year(IQR)	1.88 (0.29,7)
1 month, n(%)	12 (5.94)
2–11 months, n(%)	65 (32.18)
1–5 years old, n(%)	64 (31.68)
6–18 years old, n(%)	61 (30.20)
Clinically consistent, n(%)	198 (98.02)
Not consistent with clinical practice, n(%)	4 (1.98)
ICU, n(%)	109 (53.96)
Non-ICU, n(%)	93 (46.04)
Invasive ventilator, n(%)	39 (19.31)
Non-invasive ventilator, n(%)	65 (32.18)
Potent antibiotics, n(%)	86 (42.57)
Non-potent antibiotics, n(%)	116 (57.43)
Early mNGS, n(%)	135 (66.83)
Late mNGS, n(%)	67 (33.17)
Antibiotics adjustment, n(%)	92 (45.54)
Antibiotics without adjustment, n(%)	110 (54.46)
Extrapulmonary complications, n(%)	131 (64.85)
No complications, n(%)	71 (35.15)
Electrolyte imbalance, n	27 (13.37)
Pleural effusion, n(%)(%)	41 (20.30)
Sepsis, n(%)	24 (11.88)
Myocardial damage, n(%)	62 (30.69)
Abnormal coagulation function, n(%)	21 (10.40)
Anemia, n(%)	64 (31.68)
Liver damage, n(%)	28 (13.86)
Urinary system injury, n(%)	19 (9.41)
Cure, n(%)	148 (73.27)
Improved, n(%)	46 (22.77)
Death, n(%)	8 (3.96)

### Comparison of clinical data between intensive care unit and non-intensive care unit cases

Compared with non-ICU patients, ICU patients exhibited lower age and weight, shorter duration of cough, longer duration of dyspnoea, higher respiration and pulse rates and higher incidence of triple concave sign and crackles. The SOFA and APACHE II scores were also higher in ICU patients than in non-ICU patients. Additionally, ICU patients had lower levels of haemoglobin and albumin and higher levels of CRP, PCT, LDH, IL-6 and ALT. The number of positive bacterial cultures, restrictive antibiotic applications, combinations of >2 antibiotics and positive antibiotic-resistant genes were all higher in ICU patients than in non-ICU patients. The mNGS results indicated that ICU patients needed to receive more antibiotic upgrades than non-ICU patients, although fewer had the same level of antibiotic conversions. The ICU patients also had a higher incidence of extrapulmonary complications, cardiovascular injury and anaemia than the non-ICU patients. The statistical analysis with a significant difference (*p* < 0.05) is presented in **Table 2**.

**Table 2:** Comparison of clinical data between ICU and non-ICU patients.

Index	Total (n=202)	ICU (n=109)	Non-ICU (n=93)	Z/χ2	P
Age, year,median(IQR)	1.88 (0.29, 7)	1.17 (0.25, 6)	3.42 (0.33, 7)	2.00	0.046
Male, n(%)	129(63.86)	68 (62.39)	61 (65.59)	0.55	0.460
Fever, Days, Median (IQR)	2 (0, 6)	2 (0, 5)	3 (0, 7)	1.34	0.180
Cough, Days, Median (IQR)	4 (2, 10)	3 (0.5, 6)	7 (3, 15)	4.48	0.000
Dyspnea, Days, Median (IQR)	0 (0, 1)	0.5 (0, 2)	0 (0, 0.5)	4.75	0.000
Body temperature, °C	36.8 (36.5, 37.33)	36.8 (36.5, 37.4)	36.8 (36.5, 37.2)	1.11	0.267
Heart Rate, times/minute, Median (IQR)	132 (112, 158)	141 (120, 161)	125 (104, 137)	4.27	0.000
Respiratory Rate, times/minute, Median (IQR)	32 (25, 44.5)	38 (28, 50)	29.5 (23, 37)	4.07	0.000
Weight, kg, Median (IQR)	12 (7.5, 24.75)	10.5 (6.4, 19.5)	15 (7.6, 28)	2.68	0.007
Cyanosis, n(%)	35 (17.33)	24 (22.02)	11 (11.83)	3.64	0.056
Three concave signs, n(%)	98(48.51)	64 (58.72)	34 (36.56)	9.86	0.020
Crackles, n(%)	132 (65.35)	79 (72.48)	53 (56.99)	5.32	0.021
Stridor, n(%)	74 (36.63)	44 (40.37	39 (41.94)	2.36	0.310
White Blood Cell, ×10^9	10.18 (6.75, 13.59)	10 (6.47, 12.96)	10.72 (7, 13.95)	1.02	0.308
Neutrophil ratio, %	60.55 (42.10, 78.03)	60.9 (46.1, 77.9)	59.7 (35.9, 77.3)	1.22	0.222
Lymphocyte ratio, %	33.45 (16.35, 47.50)	34 (17.2, 44.6)	33.2 (16.4, 57.2)	1.27	0.204
Platelets, ×10^9, Median (IQR)	341.50 (244.75, 436.75)	346 (236, 448)	333 (283, 417)	0.11	0.915
Hemoglobin, g/L, Median (IQR)	111.50(97.75,123.25)	105 (93, 119)	118 (107, 128)	3.87	0.000
CRP, mg/L, Median (IQR)	8(1.02, 37.96)	10.2 (1.74, 47.6)	7.69 (1, 17.28)	2.24	0.025
PCT, ng/ml, Median (IQR)	0.3 (7.16, 0.71)	0.39 (0.19, 1.3)	0.3 (0.12, 0.33)	3.32	0.001
LDH, U/L, Median (IQR)	357(267, 669.75)	426 (269, 726)	293 (255, 469)	2.75	0.006
Interleukin 6, pg/ml, Median (IQR)	8(5.68, 25.1)	9.12 (8, 34.98)	8 (4.70, 15.87)	2.29	0.022
DD, μg/ml	1(7.7, 2)	1.1 (0.8, 3.9)	1 (0.675, 2.15)	1.76	0.078
Albumin, g/L, Median (IQR)	38 (33.60, 41.93)	36.6 (32.5, 40.2)	39.9 (36.3, 43)	4.04	0.000
ALT, U/L, Median (IQR)	26 (17, 41.5)	28 (19, 47)	24 (16, 34)	2.15	0.032
Creatinine, μmol/L, Median (IQR)	22.75 (17.98, 31.5)	22.5 (17.1, 29)	25.3 (19.9, 33.2)	1.89	0.059
BNP, pg/mL, Median (IQR)	291.5 (212., 557.5)	335 (189, 724)	268 (212, 543)	0.98	0.326
SOFA, Median (IQR)	6 (2, 8)	6 (4, 80)	4 (1, 7)	3.99	0.000
APACHEII, Median (IQR)	11 (3, 15)	13 (9, 16)	6 (2, 13)	4.90	0.000
From admission to mNGS, days, Median (IQR)	3 (2, 5)	3 (2, 5)	4 (2, 6)	1.34	0.179
Early mNGS, n(%)	135 (66.83)	78 (71.56)	57 (61, 29)	2.39	0.122
Positive of antibiotic resistance gene by mNGS, n(%)	22 (10.89)	17 (15.6)	5 (5.38)	5.4	0.020
Positive of antibiotic resistance by culture, n(%)	42 (20.79)	26 (23.85)	16 (17.20)	1.35	0.250
mNGS pathogen number, Median (IQR)	3 (2, 5)	3 (2, 4)	3 (2, 5)	1.14	0.254
Pathogen number of culture, Median (IQR)	0 (0, 1)	1 (0, 1)	0 (0, 1)	1.96	0.049
mNGS positive, n(%)	199 (98.51)	107 (98.16)	92 (98.92)	0.200	0.656
CMT positive, n(%)	96 (47.52)	59 (54.13)	37 (39.78)	4.14	0.042
CMT pathogen number, Median (IQR)	1 (0, 2)	1 (0, 1)	1 (0, 1)	0.69	0.487
Antibiotic adjustments, n(%)	92(45.54)	47 (43.12)	45(48.39)	0.56	0.454
Antibiotic regimen escalation, n(%)	45 (22.28)	30 (27.52)	15 (16.13)	3.76	0.052
Antibiotic de-escalation, n(%)	22 (10.89)	13 (11.93)	9(9.68)	0.26	0.609
No change of antibiotic regimen, n(%)	25 (12.38)	4 (3.67)	21 (22.58)	16.55	0.000
Potent antibiotics, n(%)	86 (42.57)	57 (52.30)	29 (31.18)	9.15	0.002
Combinations of antibiotics, n(%)	71 (35.1 5)	45 (41.28)	26 (27.96)	3.91	0.048
Outcomes, n(%)	193 (95.54)	100 (91.97)	93 (100)	168.97	0.080
Extrapulmonary complications, n(%)	131 (64.85)	84 (77.06)	47 (50.54)	15.49	0.000
Electrolyte imbalance, n(%)	27 (13.37)	17 (15.60)	10 (10.75)	1.02	0.310
Pleural effusion, n(%)	41 (20.30)	26 (23.85)	15 (16.13)	1.85	0.170
Sepsis, n(%)	24 (11.88)	13 (11.93)	11 (11.83)	0.00	0.980
Myocardial damage, n(%)	62 (30.69)	46 (42.20)	16 (17.20)	14.74	0.000
Abnormal coagulation function, n(%)	21 (10.40)	10 (9.17)	11 (11.83)	0.38	0.540
Anemia, n(%)	64 (31.68)	42 (38.53)	22 (23.66)	5.13	0.024
Liver damage, n(%)	28 (13.86)	18 (16.51)	10 (10.75)	1.40	0.240
Urinary system injury, n(%)	19 (9.41)	14 (12.84)	5 (5.38)	3.28	0.070
Length of stay, days, Median (IQR)	13 (10, 18)	14 (11, 19)	13 (9, 17.25)	1.27	0.203

ALT, Alanine aminotransferase; APACHE, Acute Physiology and Chronic Health Evaluation; BALF, Bronchoalveolar lavage fluid; BNP, Brain natriuretic peptide; CMT, Conventional microbiological tests; CRP, C reactive protein; DD, D Dimer; ECMO, Extracorporeal membrane oxygenation; ICU, Intensive care unit; LDH, Lactate dehydrogenase; mNGS, metagenomic next generation sequencing; PCT, Procalcitonin; SOFA, Sequential organ failure assessment; WBC, White blood cells.

### Positive rates of pathogen detection compared between metagenomic next-generation sequencing and conventional microbiological techniques

**Figure 2:**
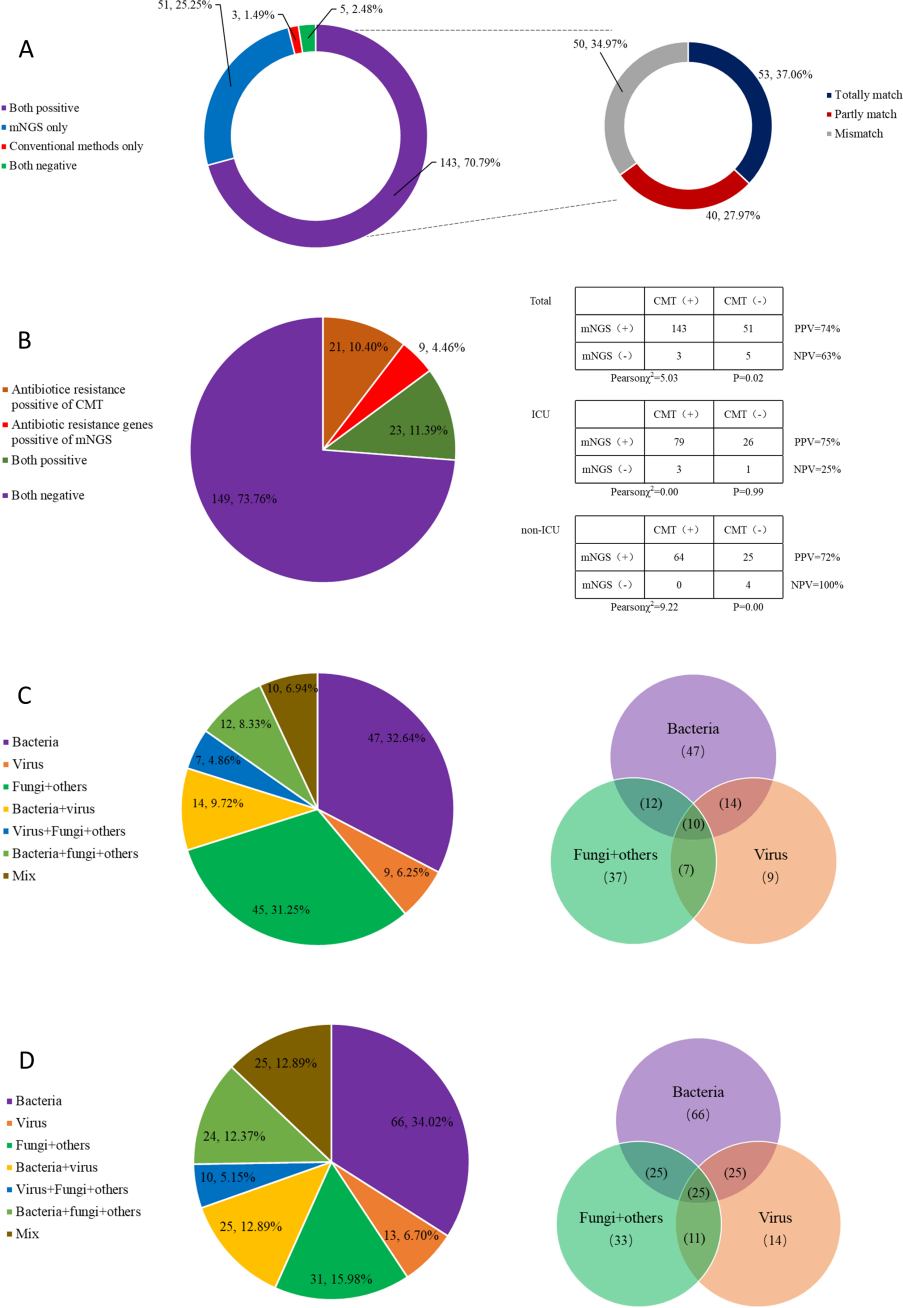
Comparison of pathogen distribution between mNGS and CMT. **(A)**. Analysis of the positive rate of microorganisms detected by mNGS and CMT and the clinical consistency analysis of positive patients; **(B)** Analysis of mNGS resistance gene positivity and bacterial culture resistance positivity; positive rate by mNGS and CMT in ICU and non-ICU groups Analysis; **(C)** CMT pathogen distribution; **(D)** mNGS pathogen distribution.

The positive rate of pathogen detection by mNGS among the 202 patients was 98.51% (199/202), which was higher than the positive rate of 47.52% (96/202) by CMT. In terms of pathogen detection, 143 patients (70.79%) tested positive by both mNGS and CMT, whereas 51 patients (25.25%) tested positive only by mNGS and 3 patients (1.49%) tested positive only by CMT; 5 patients (2.48%) tested negative by both methods (**Figure 2A**). In terms of the consistency between mNGS test results and clinical outcomes, a complete match was identified in 53 patients (37.06%), a partial match in 40 patients (27.97%) and a mismatch in 50 patients (34.97%) (**Figure 2A**). The positive predictive value (PPV) was 74% and the negative predictive value (NPV) was 63%. The Pearson chi-squared value was 5.03 with a *p*-value of 0.02. Among the ICU patients, 79 (39.11%) tested positive by both methods, 26 (12.87%) tested positive only by mNGS, 3 patients (1.49%) tested positive only by conventional methods and 1 patient (0.5%) tested negative by both methods. The PPV was 75% and the NPV was 25%. The Pearson chi-squared value was 0.00 with a *p*-value of 0.99 (**Figure 2B**). For the non-ICU patients, 64 (31.68%) tested positive by both methods, 25 (12.38%) tested positive only by mNGS and none tested positive only by conventional methods. Four patients (1.98%) tested negative by both methods. The PPV was 72% and the NPV was 100%. The Pearson chi-squared value was 9.22 with a *p*-value of 0.00 (**Figure 2B**).

Antibiotic resistance among the 202 samples was compared between these two detection methods (**Figure 2B**). A positive rate of 10.4% for antibiotic resistance was detected only by CMT, whereas a positive rate of 4.46% for antibiotic resistance genes was detected only by the mNGS. A total of 11.39% of the samples tested positive for antibiotic resistance by both methods at the same time, and 73.76% tested negative by either method.

In terms of pathogen distribution, the CMT detected bacteria in 32.64% of samples, viruses in 6.25%, fungi and other microorganisms in 31.25%, a combination of bacteria and viruses in 9.72%, a combination of viruses, fungi and others in 4.86%, a combination of bacteria, fungi and others in 8.33% and a mixture in 6.94%. On the other hand, the mNGS detected bacteria in 34.02% of samples, viruses in 6.7%, fungi and other microorganisms in 15.98%, a combination of bacteria and viruses in 12.89%, a combination of viruses, fungi and others in 5.15%, a combination of bacteria, fungi and others in 12.37% and a mixture in 12.89%. Overall, the mNGS has a higher positive rate of pathogen detection than the CMT (**Figures 2C, D**).

Interpretation Note: The PPV, NPV, and AUC values presented above were calculated using conventional microbiological techniques (CMT) as the reference standard. It is important to acknowledge that CMTs, while established in clinical practice, represent an imperfect gold standard, particularly in pretreated or critically ill patients where pathogen viability or detectability may be compromised. Consequently, these diagnostic performance metrics should be interpreted with appropriate caution, as they are inherently influenced by the limitations of the reference method against which mNGS is compared.

### Comparison of pathogens detected by metagenomic next-generation sequencing and conventional microbiological techniques

The total number of pathogens detected by mNGS was 451. *Haemophilus influenzae* accounted for 34.16% (69/202 cases), *M. pneumoniae* for 31.19% (63/202 cases), *S. pneumoniae* for 22.28% (45/202 cases), *Staphylococcus aureus* for 19.31% (39/202 cases), *Bordetella pertussis* for 10.89% (22/202 cases), *Acinetobacter baumannii* for 8.91% (18/202 cases) and *Moraxella catarrhalis* for 6.44% (13/202 cases). Additionally, the *Aspergillus* genus was found in 13.86% (28/202 cases), *Pneumocystis jiroveci* in 9.9% (20/202 cases), *Candida albicans* and human herpesvirus type 5 in 8.91% each (18/202 cases), pantoviru*s* in 6.44% (13/202 cases), human herpesvirus 7 in 5.45% (11/202 cases), human herpesvirus 1 in 4.95% (10/202 cases) and influenza A and B viruses and RSV each contributed to 1.49% of cases (3/202 cases).

**Figure 3:**
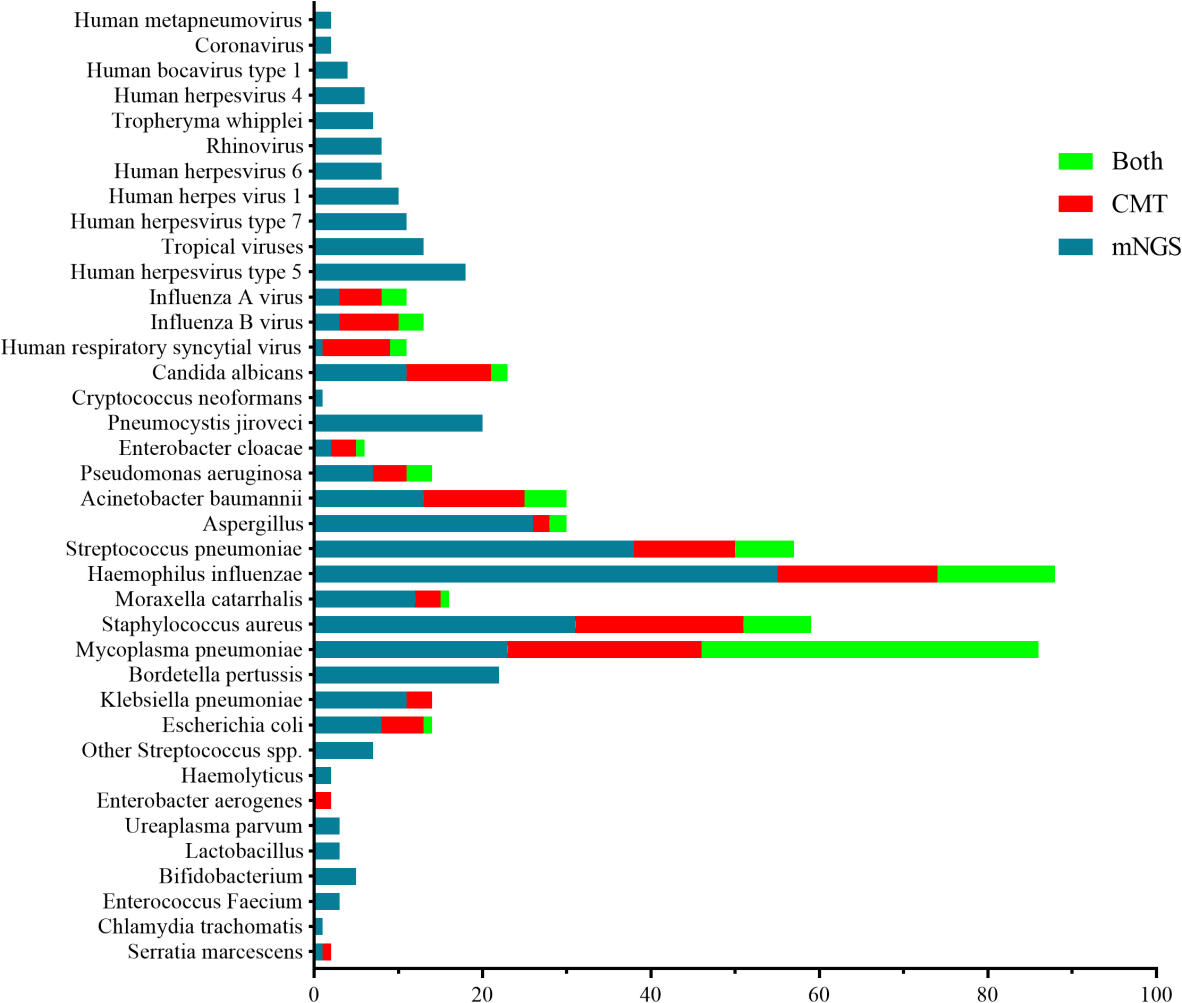
Number of pathogens detected by mNGS and/or CMT in the 202 BALF samples. Note: “Both”: Represents pathogens detected by both metagenomic next-generation sequencing (mNGS) and conventional microbiological techniques (CMT). These pathogens are identified by both detection methods, indicating a high degree of consistency in their presence in the samples. “CMT”: Indicates pathogens detected only by conventional microbiological techniques (such as bacterial culture, PCR, etc.) but not by mNGS. “mNGS”: Refers to pathogens detected only by mNGS but not by CMT.

The CMT detected a total of 226 pathogens. M*ycoplasma pneumoniae* was detected in 63 of 202 cases (31.19%), *S. aureus* accounted for 28 cases (13.86%), *H. influenzae* for 33 cases (16.34%), *S. pneumoniae* for 19 cases (9.41%), *A. baumannii* for 17 cases (8.42%), *Escherichia coli* for 6 cases (2.97%), *C. albicans* for 12 cases (5.94%) and *Aspergillus* for 4 cases (1.98%). Additionally, RSV, influenza B virus and influenza A virus accounted for 4.95%, 4.95% and 3.96%, respectively. *Mycoplasma pneumoniae* accounted for 40 cases (19.8%), *H. influenzae* for 14 cases (4.95%), *S. aureus* for 8 cases (3.96%) and *S. pneumoniae* for 7 cases (3.47%). Influenza B virus, influenza A virus, RSV, *Aspergillus* and *C. albicans* each accounted for 3 cases (1.49%), and *P. jiroveci* accounted for 2 cases (0.99%) (**Figure 3**).

Overall, the major bacterial pathogens identified in this study were *H. influenzae*, *S. pneumoniae* and *S. aureus*. The major viral pathogens were the influenza virus, human herpesvirus 5, serovirus and human RSV. The main fungal pathogens included *Aspergillus* spp., *C. albicans* and *P. jiroveci*; *M. pneumoniae* and *B. pertussis* were also commonly detected.

### Clinical correlation of mNGS-only positive cases

Among the cases that were positive by mNGS but negative by conventional microbiological tests (CMT), we performed comprehensive clinical correlation to assess the validity of mNGS findings. In the “mNGS-only” cases, targeted antimicrobial therapy was initiated based on the mNGS results, and clinical improvement—defined as resolution of fever, reduction in inflammatory markers (e.g., CRP, procalcitonin), and/or radiological improvement on follow-up imaging—was observed within 72–96 hours. Additionally, pathogen-specific PCR assays were available for 18 cases (e.g., for Mycoplasma pneumoniae, influenza virus, adenovirus, and Pneumocystis jirovecii), and 16 (88.9%) showed concordant results with mNGS. These findings support the clinical relevance and diagnostic reliability of mNGS in detecting pathogens missed by CMT.

### The timing of metagenomic next-generation sequencing and the impact of antibiotic adjustment based on its outcomes

Analysis of the impact of the timing of mNGS on outcomes revealed that early mNGS resulted in shorter ICU and hospitalisation days than late mNGS. This aligns with the principle of targeted therapy, as precise pathogen identification could minimise unnecessary drug exposure. The mNGS results guided changes in the antibiotic treatment regimen for many children (92/202). Among them, 110 cases (54.46%) did not require adjustment, 45 cases (22.28%) needed an antibiotic regimen escalation, 22 cases (10.89%) required an antibiotics regimen de-escalation and 25 cases (12.38%) were adjusted to the same level.

In the early mNGS group, patients who had antibiotic adjustments had lower instances of liver and urinary tract damage than those who did not have adjustments (*p* = 0.04), indicating that timely adjustments may reduce organ toxicity. This aligns with the principle of targeted therapy, as precise pathogen identification could minimise unnecessary drug exposure. The early mNGS group had a shorter hospitalisation duration than the late mNGS group (median 13 vs. 15 days, log-rank p = 0.013), while the use of potent antibiotics did not differ significantly between the two groups, suggesting that the benefit of early testing is attributable to timely pathogen-directed therapy rather than greater disease severity. In the late mNGS group, patients who did not have antibiotic adjustments had lower rates of coagulation function abnormalities and reduced usage of advanced antibiotics than those who did adjustments (*p* = 0.03, 0.01) (**Table 3**), highlighting that delayed testing may limit opportunities for appropriate de-escalation, leading to prolonged broad-spectrum use. Overall, the successful application of mNGS in diagnosing and treating childhood infections may significantly improve prognosis.

**Table 3:** The impact of antibiotic adjustment on the outcome of mNGS.

Variable	Early mNGS (n =135)				Late mNGS(n=67)			
	Total	No adjustment (n=73)	Adjustment (n=62)	P	Total	No adjustment (n=32)	Adjustment (n=35)	P
Good outcomes,n(%)	129(95.56)	68(93.2)	61(98.4)	0.14	65(97.01)	31(96.9)	34(97.1)	0.95
Extrapulmonary complications,n(%)	94(69.63)*	48(65.8)	46(74.2)	0.13	37(55.22)*	15(46.9)	22(62.9)	0.19
Electrolyte imbalance,n(%)	19(14.1)	11(15.1)	8(12.9)	0.71	8(11.9)	4(12.5)	4(11.4)	0.89
Pleural effusion,n(%)	31(23)	17(23.33)	14(22.6)	0.92	10(14.9)	6(18.8)	4(11.4)	0.40
Sepsis,n(%)	17(12.6)	7(9.6)	10(16.1)	0.25	7(10.4)	2(6.3)	5(14.3)	0.28
Myocardial damage,n(%)	45(33.3)	21(28.8)	24(38.7)	0.22	17(25.4)	10(31.3)	7(20)	0.29
Abnormal coagulation function,n(%)	14(10.4)	9(12.3)	5(8.1)	0.42	7(10.4)	1(3.1)	7(20)	0.03
Anemia,n(%)	41(30.4)	24(32.9)	17(27.4)	0.49	23(34.3)	10(31.3)	13(37.1)	0.61
Liver damage,n(%)	22(16.3)	8(11.0)	14(22.6)	0.04	6(9)	1 (3.1)	5(14.3)	0.11
Urinary system damage,n(%)	14(10.4)	4(5.5)	10(16.1)	0.04	5(7.5)	2(6.3)	3(8.6)	0.72
Invasive ventilation, days, Median (IQR)	0	0	0	0.28	0	0(0,1.5)	0	0.14
Non-invasive ventilation, days, Median (IQR)	0(0,4)	0(0,2)	0(0,4)	0.75	0(0,5)	0(0,4)	0(0,4.5)	0.43
ICU days, Median (IQR)	2(0,8)#	1.5(0,9.75)	2(0,7)	0.41	7(0,11)#	7(0,13.5)	6(0,10)	0.23
Length of stay, Median (IQR)	13(10,17)**	14(11,20)	9(12,14)	0.003	15(12,21)**	15(13,24)	14.5(11,18)	0.21
Potent antibiotics,n(%)	54(40)	25(34.24)	32(51.61)	0.04	32(47.76)	10(31.3)	22(62.9)	0.01
Combinations of antibiotics, n(%)	49(36.3)	23(31.5)	26(41.9)	0.21	21(31.34)	8(25)	13(37.1)	0.28

Explanation: In the table, *P<0.05, **P<0.01, #*P*<0.001,all suggesting that these data have significant differences statistically between two groups.

### Antibiotics resistance genes detected by metagenomic next-generation sequencing

**Figure 4:**
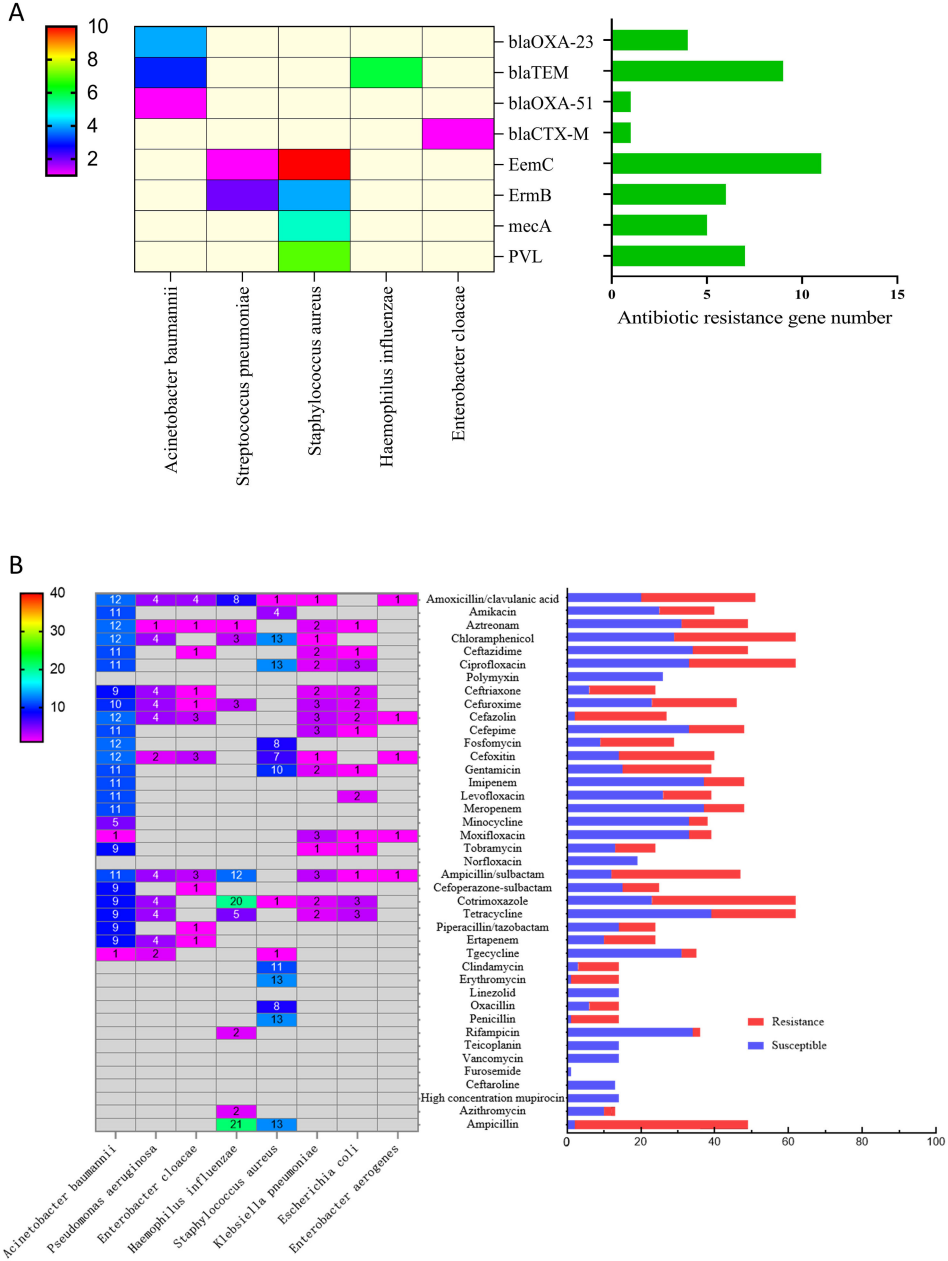
AMR genes and antibiotics resistance rate. **(A)** left panel: heatmap of AMR gene detected by mNGS, right panel: count of AMR genes detected by mNGS. **(B)** left panel: heatmap of antibiotics resistance rate by PST, right panel: PST result for each antibiotic.

A total of five antibiotic resistance genes were identified using mNGS. The most frequently detected gene was ermC, associated with resistance to *S. aureus*. Genes such as ermB (6/44) and ermC (11/44), linked to macrolide and lincosamide antibiotic resistance in *S. aureus* and *S. pneumoniae*, were less commonly detected. Additionally, the PVL gene, associated with *S. aureus* virulence (7/44), and the blaTEM gene, related to ESBL-mediated cephalosporin resistance, were found in 6 cases of *H. influenzae* (6/44) and 3 cases of *A. baumannii* (3/44), respectively. The mecA gene, responsible for oxacillin- or methicillin-resistant *S. aureus* (5/44), and the blaOXA-23 gene, associated with carbapenem resistance in *A. baumannii* (4/44), were also detected. Last, the blaCTX-M (1/44), blaOXA-51 (1/44) and blaTEM (9/44) genes were identified, which are linked to penicillin and cephalosporin resistance in various bacterial species (**Figure 4A**).

Interpretation Note: The detection of antibiotic resistance genes by mNGS indicates their presence within the microbial genetic material in the sample. However, it is crucial to emphasize that the presence of a resistance gene does not always equate to phenotypic antibiotic resistance in the pathogen. Gene expression levels, the presence of functional regulatory elements, and bacterial physiological state can influence whether a detected gene confers actual resistance to treatment.

### Antibiotic resistance detected by conventional microbiological techniques

The antibiotics with the highest resistance rates are ampicillin (94.44%), erythromycin (92.86%), penicillin (92.86%), cefazolin (92.6%), clindamycin (78.57%), ceftriaxone (75%) and ampicillin/sulbactam (74.47%). Additionally, the top antimicrobial resistance rates of enzyme inhibitors are associated with amoxicillin/clavulanic acid (60.78%), piperacillin/tazobactam (41.67%) and cefoperazone–sulbactam (40%). The resistance rates associated with third-generation or above cephalosporins are cefepime (31.25%), ceftazidime (30.61%), ceftriaxone (22.92%) and cefoperazone–sulbactam (40%). Carbapenem resistance rates are imipenem (22.92%) and meropenem (22.92%). No resistance to linezolid and vancomycin has yet been found. The resistance rate of *A. baumannii* to carbapenem antibiotics is 91.67% (11/12), to aminoglycoside antibiotics is 9.68% (3/31), to third-generation cephalosporin antibiotics is 91.67% (11/12) and to quinolones is 67.65% (23/34). In contrast, *Pseudomonas aeruginosa* shows low resistance rates to these four types of antibiotics, specifically 33.33% (4/12), 0%, 33.33% (4/12) and 0%, respectively. The antibiotic resistance rate of *Klebsiella pneumoniae* also varies significantly, with 0% for carbapenems, 20% (2/10) for aminoglycosides and 20% (2/10) for third-generation cephalosporins. The average resistance rate to bacteriocins was 33.33% (4/12) and to quinolones was 41.67% (5/12). Resistance status of *S. aureus*: aminoglycosides 51.85% (14/27), penicillins 86.96% (20/23), macrolides 7.69% (1/13), tetracyclines 0%, clindamycin 78.57% (11/14), chloramphenicol 100%, ciprofloxacin 100%, ampicillin 92.86% (13/14), clindamycin 78.57% (11/14), fosfomycin 61.54% (8/13) and cefoxitin 53.85% (7/13). Resistance status of *H. influenzae*: ampicillin 95.45% (21/22), cotrimoxazole 90.91% (20/22), ampicillin/sulfamethoxazole Bactam 54.55% (12/22), amoxicillin/clavulanic acid 36.37% (8/22), tetracycline 22.73% (5/22) and chloramphenicol 13.64% (3/22). Resistance to carbapenem antibiotics is mainly concentrated in *A. baumannii*, and resistance to aminoglycoside antibiotics is mainly concentrated in *A. baumannii* and *S. aureus*. Tetracyclines show high sensitivity to *S. aureus*, *Enterobacter cloacae and H. influenzae*. Quinolones are highly sensitive to *P. aeruginosa, H. influenzae, S. aureus* and *E. cloacae* (**Figure 4B**).

### The impact of metagenomic next-generation sequencing timing and antibiotic adjustment on length of stay

**Figure 5:**
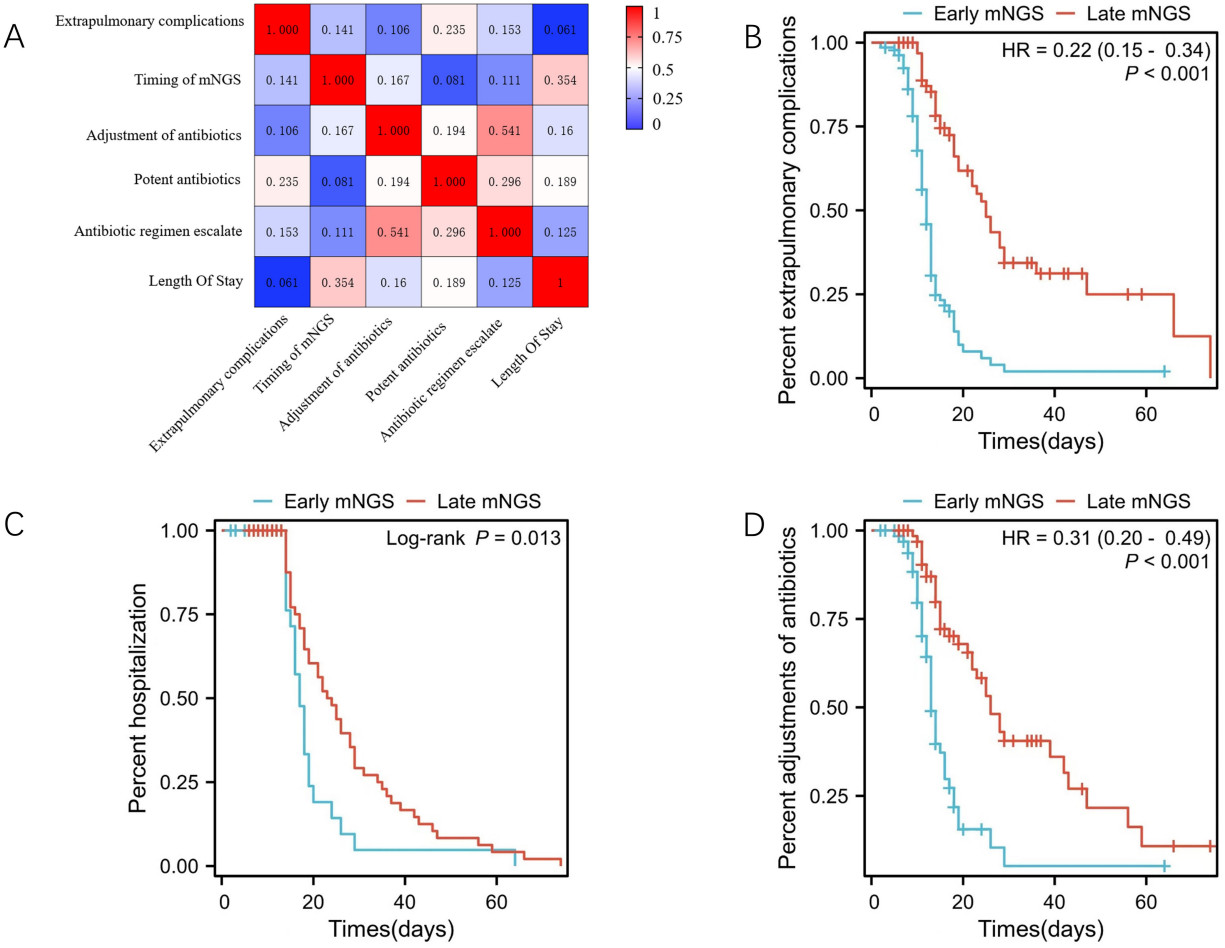
Analysis of the effect of mNGS time on treatment plan adjustment and hospitalization outcome. **(A)** Correlation of the effect of mNGS time on antibiotic treatment adjustments and hospitalization outcome. **(B)** The effect of early mNGS on the incidence of extrapulmonary complications, HR = 0.22(0.15-0.34), P<0.001. **(C)** effect of early mNGS on hospital stay, Log-rank,P=0.013. **(D)** effect of early mNGS on antibiotic adjustment, HR = 0.31(0.20-0.49), P<0.001.

The timing of mNGS showed a weak positive correlation with extrapulmonary complications (*r* = 0.141, *p* = 0.046) and antibiotic adjustment (*r* = 0.167, *p* = 0.017). Notably, these correlations translated to tangible differences in LOS; early mNGS was associated with a median LOS of 13 days compared with 15 days in the late group (log-rank, *p* = 0.013), confirming that earlier testing shortens hospitalisation (**Figure 5**).

The use of potent antibiotics was moderately correlated with extrapulmonary complications (*r* = 0.235, *p* = 0.001), suggesting that broad-spectrum agents may not fully prevent complications and could reflect more severe disease. Antibiotic escalation was weakly correlated with extrapulmonary complications (*r* = 0.153, *p* = 0.030), likely because escalation is often triggered by persistent infection, which itself increases complication risk. There was a weak positive correlation between antibiotic adjustment and the use of potent antibiotics (*r* = 0.194, *p* = 0.006) and a strong positive correlation between antibiotic adjustment and antibiotic escalation (*r* = 0.541, *p* < 0.001), indicating that adjustments frequently involve stepping up therapy, though de-escalation remains clinically meaningful.

Survival analysis further confirmed that early mNGS reduced extrapulmonary complications (hazard ratio [HR] = 0.22, 95%CI: 0.15–0.34, *p* < 0.001) and increased early antibiotic adjustment rates (HR = 0.31, 95%CI: 0.20–0.49, *p* < 0.001), both of which are key drivers of shorter LOS. Together, these findings demonstrate that early mNGS facilitates timely, targeted antibiotic adjustments, thereby reducing complications and shortening hospital stays.

## Discussion

In this study, we conducted a comprehensive evaluation of 202 BALF samples from paediatric patients with pneumonia aged 1 month to 18 years who underwent mNGS. Our analysis focused on assessing the diagnostic performance of mNGS and its potential role in guiding antibiotic application. Our results indicated that mNGS was highly effective in detecting pathogens and valuable for guidance for antimicrobial treatment, ultimately improving clinical management.

### Diagnostic performance of metagenomic next-generation sequencing

In these samples, the top pathogens detected by mNGS in this study were consistent with a previous study conducted by our research group. However, the time required to obtain the results was shorter (Wu et al., 2023). The positive rate of mNGS was higher than that of CMT, with a PPV of 74% and an NPV of 63% overall. In ICU patients, the PPV of mNGS was slightly higher at 75%, but the NPV of mNGS was lower at 25%. This indicates that mNGS demonstrates relatively lower specificity in pathogen detection. However, this does not imply that pathogens identified solely through mNGS are false positives. The significantly lower NPV of mNGS in ICU patients compared with non-ICU patients is likely influenced by the complexity of infections, higher pathogen loads and the critical condition of ICU patients (Zhao et al., 2023; Yao et al., 2024). Pathogens identified by mNGS could potentially provide valuable clinical insights (Zhu et al., 2023). For non-ICU patients, the PPV of mNGS was slightly lower at 72%, but the NPV was 100%, indicating that negative mNGS results are highly reliable. These findings are consistent with a study by Chen et al. that reported mNGS had a higher detection rate of *P. jiroveci* with a PPV of 89.5% and an NPV of 100% compared with traditional methods (Chen et al., 2024). The mNGS showed a PPV value of 60.0% and an NPV value of 94.1% for bacterial detection. Dong et al. also found high sensitivity and specificity of mNGS (Tao et al., 2022; Dong et al., 2023).

Specifically, an antibiotic resistance gene was detected in 22 out of 202 samples (10.89%) using mNGS. In contrast, traditional culture methods detected antibiotic resistance in 42 out of 202 samples (20.79%). This suggests that traditional culture methods may be more sensitive in detecting antibiotic resistance or that they may detect different types of resistance not picked up by mNGS. Due to the high proportion of host nucleic acids in clinical samples, the bacterial content in the original sequencing data of mNGS is relatively low. This can affect the detection of resistance genes/mutations by mNGS (Gan et al., 2021). However, it is important to note that these methods may be complementary, as mNGS can detect resistance genes even when the bacteria are not currently expressing them, whereas culture methods can only detect resistance when the bacteria are actively resisting the antibiotics (Serpa et al., 2022; Liang et al., 2024).

It is important to distinguish between the detection of a microbial nucleic acid sequence by mNGS and the clinical diagnosis of an active infection caused by that organism. This distinction is particularly relevant for organisms such as fungi (e.g., Aspergillus spp., Candida albicans) and herpesviruses (e.g., HHV-5, HHV-7), which were identified in a subset of our patients. These microorganisms can represent colonization, environmental contamination, latent viral reactivation of uncertain clinical significance, or true pathogenic drivers of disease. The high sensitivity of mNGS increases the likelihood of detecting these sequences. Therefore, the clinical relevance of mNGS findings for such pathogens must be carefully interpreted within the full clinical context, including host immune status, radiographic features, supporting microbiological data, and the patient’s treatment response. A positive mNGS result should prompt critical assessment rather than automatic assumption of causative infection.

### Metagenomic next-generation sequencing-based antibiotic treatment adjustment

The detection of mixed infections (e.g. bacterial–viral co-infections) by mNGS highlights its clinical utility in identifying complex microbial profiles that may influence disease severity and treatment outcomes (Zheng et al., 2021). For instance, bacterial–viral co-infections (e.g. influenza virus with *S. pneumoniae*) are associated with heightened inflammatory responses and increased risk of complications such as sepsis and respiratory failure (Wei et al., 2023). The presence of mixed infections may necessitate combined antimicrobial and antiviral therapies, underscoring the importance of comprehensive pathogen detection in guiding targeted treatment (Zheng et al., 2021).

In developing countries, bacteria continue to be a significant cause of severe pneumonia (Jambo et al., 2023), necessitating prompt antibiotic treatment (Walker et al., 2022). This study also revealed that ICU patients had a higher number of positive bacterial cultures and a greater incidence of drug-resistant genes. Several studies emphasised the importance of early pathogen detection and intervention in the treatment of childhood pneumonia, particularly in the ICU setting (de Benedictis et al., 2020). Utilising mNGS to guide antibiotic therapy has the potential to enhance patient outcomes. Our study also demonstrated that early mNGS testing could lead to reduced hospital stays and fewer ICU days, which aligned with the findings of Chen et al. that early mNGS-based treatment adjustments may improve the prognosis of neonatal infections (Chen L. et al., 2022). Our study supported this standpoint, as we observed a higher likelihood of antibiotic modification with longer hospital stays and delayed mNGS testing. Additionally, the positive rate of mNGS was significantly higher than that of traditional methods, highlighting the need for early performance of mNGS testing. It is important to note that CMT, although traditionally used as a reference, have well-documented limitations in sensitivity – particularly for fastidious bacteria, intracellular pathogens and viruses – due to constraints in culture viability, sample quality and prior antibiotic use. Therefore, using CMT alone as a ‘gold standard’ may underestimate the true diagnostic accuracy of mNGS. In this study, we observed that a significant number of mNGS+/CMT− results were clinically meaningful, as evidenced by PCR confirmation, therapeutic response and serological trends. This supports the notion that mNGS can detect pathogens missed by conventional methods, especially in pre-treated or critically ill patients. However, we also acknowledge the potential for false positives due to contamination or colonisation. Thus, integrating mNGS findings with clinical context, host response and orthogonal testing (e.g. PCR) is essential for accurate interpretation.

In this study, the mNGS results showed that 50 cases (24.75%) had their antibiotics escalated, and 22 cases (10.89%) had their antibiotics de-escalated. Among ICU patients, most received escalated antibiotics and restricted use of antibiotics, whereas non-ICU patients received adjustments in sensitive antibiotics at the same level. Wei et al.’s study also changed the treatment approach for 26 children (31.0%) based on mNGS results, resulting in symptom relief (Wei et al., 2023). These findings suggested that mNGS could offer valuable guidance for adjusting antibiotic therapy to ensure patients receive the most appropriate treatment, aligning with the principles of precision medicine (Abdulla et al., 2021). Additionally, the mNGS results indicated that 109 cases (53.96%) did not require any adjustment in the antibiotic treatment plan, indicating the initial antibiotic selection was effective for these patients (Zhu et al., 2023). Patients with early mNGS experienced fewer extrapulmonary complications (log-rank *p* = 0.001), shorter hospitalisation times (log-rank *p* = 0.014) and higher rates of early antibiotic adjustment (log-rank *p* = 0.005) than those with late mNGS. This study emphasised the significance of early detection and intervention of infectious agents, particularly in the ICU setting, for the treatment of severe pneumonia in children.

To exclude confounding factors other than mNGS, we performed subgroup analyses considering physician seniority (≥5 years vs <5 years) and found no significant difference in antibiotic adjustment rates between groups (46.2% vs 45.8%, *p* = 0.94). Multivariate regression also confirmed that mNGS timing remained an independent predictor of outcomes after adjusting for age, disease severity and comorbidities (HR = 0.31, 95%CI: 0.20–0.49, *p* < 0.001). These results suggest that mNGS itself, rather than physician experience or baseline health status, drives the observed improvements in clinical outcomes.

### Differences between intensive care unit and non-intensive care unit patients: causes and clinical implications

Our analysis revealed significant differences between ICU and non-ICU patients, including higher rates of extrapulmonary complications, more frequent antibiotic escalation and worse inflammatory profiles in ICU patients (Sun et al., 2022). These disparities warrant further exploration of their underlying causes and clinical implications.

First, the higher complication rate in ICU patients is likely driven by their more severe baseline condition, as evidenced by higher SOFA and APACHE II scores, elevated inflammatory markers (CRP, PCT, IL-6) and lower albumin levels (Wu et al., 2025). These factors indicate a more aggressive disease course and compromised host immunity, which independently increase the risk of organ dysfunction and secondary infections (de Benedictis et al., 2020; Rueda et al., 2022). Notably, the timing of mNGS application (early vs late) did not differ significantly between ICU and non-ICU groups (*p* = 0.122), suggesting that the observed differences in complications are primarily related to disease severity rather than mNGS usage.

Second, the higher frequency of antibiotic escalation in ICU patients may reflect the greater prevalence of drug-resistant pathogens (detected via both mNGS and CMT) and mixed infections in this cohort (Zhang et al., 2024). For example, ICU patients had a higher positive rate of antibiotic resistance genes (15.6% vs 5.38%, *p* = 0.020) and more frequent use of combination antibiotics (41.28% vs 27.96%, *p* = 0.048), which aligns with the need for broader-spectrum coverage in severe infections. However, mNGS still played a critical role in guiding these adjustments; 44.95% of ICU patients underwent antibiotic changes based on mNGS results, with 32.11% requiring escalation due to detection of resistant pathogens (e.g. *S. aureus* carrying mecA).

Clinically, these findings emphasise that ICU patients may derive particular benefit from early mNGS testing. Despite their more severe illness, timely pathogen identification via mNGS could help optimise antibiotic selection, potentially reducing unnecessary escalation and limiting complications. For instance, in our cohort, ICU patients with early mNGS had a non-significant trend toward fewer extrapulmonary complications compared with those with late mNGS (72.1% vs 81.3%, *p* = 0.18), suggesting that earlier intervention may mitigate the impact of baseline severity.

## Limitations

This study has several limitations that should be considered. It is a single-centre retrospective study, and all participants were from the First Affiliated Hospital of Xinxiang Medical University. This may lead to selection bias, and the sample may not be widely representative, thus limiting the external validity of the research results. To address this, we performed multivariate regression adjusting for age, disease severity and inflammatory markers. The sample size of 202 patients may not be large enough to detect certain effects or ensure that the sample is representative of the broader population. The timing of mNGS, which showed correlations with various outcomes, could introduce bias if it were decided based on factors other than clinical judgment. Future research would focus on prospective studies to minimise potential recording bias and confounding factors. Additionally, multicentre studies with larger sample sizes could be conducted to validate the effectiveness of mNGS in the paediatric population, and further investigations could be performed to understand the role of drug resistance gene positivity and bacterial culture resistance in treatment decisions. Furthermore, our long-term follow-up focused primarily on clinical recurrence of pneumonia and did not include objective measures of pulmonary function or patient-reported quality of life. While recurrence is an important outcome, it does not capture potential subclinical sequelae, such as airway remodelling, reduced exercise tolerance or persistent respiratory symptoms, which may affect daily functioning. The absence of standardised lung function testing – particularly in younger children who are often unable to perform spirometry reliably – and the logistical challenges of administering validated paediatric quality-of-life instruments across diverse age groups limited our ability to collect these data. As a result, the full impact of SCAP on long-term respiratory health and well-being remains incompletely characterised in this cohort. Future multicentre studies should aim to incorporate structured pulmonary function assessments and age-appropriate quality-of-life metrics to enable a more comprehensive evaluation of post-SCAP outcomes.

This study employed a single-centre retrospective design, which inherently carries a risk of selection bias. Although we used multivariate regression models to adjust for key confounding variables – including age, disease severity (SOFA and APACHE II scores), inflammatory markers (CRP, PCT), comorbidities, underlying immune status and evidence of concurrent viral infections – we acknowledge that residual or unmeasured confounding may persist. For instance, detailed immunological profiles, nutritional status, prior antibiotic exposure and socioeconomic factors were not fully captured in the electronic medical records and thus could not be adjusted for. As a result, the observed associations should be interpreted with caution, particularly regarding causal inference. Furthermore, the external validity of our findings may be limited due to the homogeneous patient population from a single tertiary care centre. Future prospective multicentre studies with standardised data collection are needed to validate these results in more diverse paediatric populations and to better account for potential confounders.

Future research directions should include the following: (1) prospective multicentre trials with stratified randomisation by age and disease severity to validate mNGS efficacy in diverse paediatric populations; (2) cost-effectiveness analyses comparing mNGS with conventional methods, particularly in resource-limited settings; (3) longitudinal studies (≥1 year) to assess the impact of mNGS on long-term outcomes, such as lung function and antibiotic resistance rates; (4) integration of mNGS with host immune profiling to improve pathogen identification and treatment response prediction; (5) development of standardised protocols for mNGS implementation in paediatric SCAP management, including optimal timing and interpretation criteria for mixed infections.

## Conclusion

The study concludes that mNGS of BALF is a valuable tool in diagnosing and managing SCAP in children. The mNGS method showed a higher positive rate than CMT. The study found that the timing of mNGS was associated with extrapulmonary complications and antibiotic adjustment. Early mNGS was associated with fewer extrapulmonary complications, shorter hospital stays and higher rates of early antibiotic adjustment than late mNGS. These findings suggest that mNGS can guide the selection and adjustment of antibiotics, potentially contributing to reduced antibiotic resistance.

## Data Availability

The original contributions presented in the study are included in the article/supplementary material. Further inquiries can be directed to the corresponding authors.

## References

[B1] Abdulla A. Edwina E. E. Flint R. B. Allegaert K. Wildschut E. D. Koch B. C. P. . (2021). Model-informed precision dosing of antibiotics in pediatric patients: A narrative review. Front. Pediatr. 9, 624639. doi: 10.3389/fped.2021.624639, PMID: 33708753 PMC7940353

[B2] Alpert M. S. Htwe Y. M. (2024). Flexible bronchoscopy and bronchoalveolar lavage (BAL). J. Med. Insight 2024. doi: 10.24296/jomi/448

[B3] Bradley J. S. Byington C. L. Shah S. S. Alverson B. Carter E. R. Harrison C. . (2011). The management of community-acquired pneumonia in infants and children older than 3 months of age: clinical practice guidelines by the Pediatric Infectious Diseases Society and the Infectious Diseases Society of America. Clin. Infect. Dis. 53, e25–e76. doi: 10.1093/cid/cir531, PMID: 21880587 PMC7107838

[B4] Chen S. Kang Y. Li D. Li Z . (2022). Diagnostic performance of metagenomic next-generation sequencing for the detection of pathogens in bronchoalveolar lavage fluid in patients with pulmonary infections: Systematic review and meta-analysis. Int. J. Infect. Dis. 122, 867–873. doi: 10.1016/j.ijid.2022.07.054, PMID: 35907477

[B5] Chen W. Liu G. Cui L. Tian F. Zhang J. Zhao J. . (2024). Evaluation of metagenomic and pathogen-targeted next-generation sequencing for diagnosis of meningitis and encephalitis in adults: A multicenter prospective observational cohort study in China. J. Infect. 88, 106143. doi: 10.1016/j.jinf.2024.106143, PMID: 38548243

[B6] Chen L. Zhao Y. Wei J. Huang W. Ma Y. Yang X. . (2022). Metagenomic next-generation sequencing for the diagnosis of neonatal infectious diseases. Microbiol. Spectr. 10, e0119522. doi: 10.1128/spectrum.01195-22, PMID: 36409152 PMC9769891

[B7] Chhangani N. P. Amandeep M. Choudhary S. Gupta V. Goyal V . (2015). Role of acute physiology and chronic health evaluation II scoring system in determining the severity and prognosis of critically ill patients in pediatric intensive care unit. Indian J. Crit. Care Med. 19, 462–465. doi: 10.4103/0972-5229.162463, PMID: 26321805 PMC4548415

[B8] CLSL (2022). Performance standards for antimicrobial susceptibility testing, M100. 32st ed (Wayne, PA.: Clinical and Laboratory Standards Institute).

[B9] Collaborators G L (2022). Age-sex differences in the global burden of lower respiratory infections and risk factors, 1990-2019: results from the Global Burden of Disease Study 2019. Lancet Infect. Dis. 22, 1626–1647. doi: 10.1016/S1473-3099(22)00510-2, PMID: 35964613 PMC9605880

[B10] de Benedictis F. M. Kerem E. Chang A. B. Colin A. A. Zar H. J. Bush A. . (2020). Complicated pneumonia in children. Lancet 396, 786–798. doi: 10.1016/S0140-6736(20)31550-6, PMID: 32919518

[B11] Dong Y. Chen Q. Tian B. Li J. Li J. Hu Z. . (2023). Advancing microbe detection for lower respiratory tract infection diagnosis and management with metagenomic next-generation sequencing. Infect. Drug Resist. 16, 677–694. doi: 10.2147/IDR.S387134, PMID: 36743335 PMC9896973

[B12] Faro A. Wood R. E. Schechter M. S. Leong A. B. Wittkugel E. Abode K. . (2015). American Thoracic Society *Ad Hoc* Committee on Flexible Airway Endoscopy in Children. Official American Thoracic Society technical standards: flexible airway endoscopy in children. Am. J. Respir. Crit. Care Med. 191, 1066–1080. doi: 10.1164/rccm.201503-0474ST, PMID: 25932763

[B13] Gan M. Wu B. Yan G. Li G. Sun L. Lu G. . (2021). Combined nanopore adaptive sequencing and enzyme-based host depletion efficiently enriched microbial sequences and identified missing respiratory pathogens. BMC Genomics 22, 732. doi: 10.1186/s12864-021-08023-0, PMID: 34627155 PMC8501638

[B14] Hu X. Zhao Y. Han P. Liu S. Liu W. Mai C. . (2023). Novel Clinical mNGS-Based Machine Learning Model for Rapid Antimicrobial Susceptibility Testing of Acinetobacter baumannii. J. Clin. Microbiol. 61, e0180522. doi: 10.1128/jcm.01805-22, PMID: 37022167 PMC10204632

[B15] Jambo A. Gashaw T. Mohammed A. S. Edessa D . (2023). Treatment outcomes and its associated factors among pneumonia patients admitted to public hospitals in Harar, eastern Ethiopia: a retrospective follow-up study. BMJ Open 13, e065071. doi: 10.1136/bmjopen-2022-065071, PMID: 36792331 PMC9933768

[B16] Larsson D. G. J. Flach C. F. (2022). Antibiotic resistance in the environment. Nat. Rev. Microbiol. 20, 257–269. doi: 10.1038/s41579-021-00649-x, PMID: 34737424 PMC8567979

[B17] Liang W. Zhang Q. Qian Q. Wang M. Ding Y. Zhou J. . (2024). Diagnostic strategy of metagenomic next-generation sequencing for gram negative bacteria in respiratory infections. Ann. Clin. Microbiol. Antimicrob. 23, 10. doi: 10.1186/s12941-024-00670-x, PMID: 38302964 PMC10835912

[B18] Lin T. Tu X. Zhao J. Huang L. Dai X. Chen X. . (2023). Microbiological diagnostic performance of metagenomic next-generation sequencing compared with conventional culture for patients with community-acquired pneumonia. Front. Cell Infect. Microbiol. 13, 1136588. doi: 10.3389/fcimb.2023.1136588, PMID: 37009509 PMC10061305

[B19] Matics T. J. Sanchez-Pinto L. N. (2017). Adaptation and validation of a pediatric sequential organ failure assessment score and evaluation of the sepsis-3 definitions in critically ill children. JAMA Pediatr. 171, e172352. doi: 10.1001/jamapediatrics.2017.2352, PMID: 28783810 PMC6583375

[B20] Nascimento-Carvalho C. M. (2020). Community-acquired pneumonia among children: the latest evidence for an updated management. J. Pediatr. (Rio J) 96 Suppl 1, 29–38. doi: 10.1016/j.jped.2019.08.003, PMID: 31518547 PMC7094337

[B21] Qu J. Zhang J. Chen Y. Huang Y. Xie Y. Zhou M. . (2022). Aetiology of severe community acquired pneumonia in adults identified by combined detection methods: a multi-centre prospective study in China. Emerg. Microbes Infect. 11, 556–566. doi: 10.1080/22221751.2022.2035194, PMID: 35081880 PMC8843176

[B22] Rueda Z. V. Aguilar Y. Maya M. A. López L. Restrepo A. Garcés C. . (2022). Etiology and the challenge of diagnostic testing of community-acquired pneumonia in children and adolescents. BMC Pediatr. 22, 169. doi: 10.1186/s12887-022-03235-z, PMID: 35361166 PMC8968093

[B23] Schramm D. Freitag N. Nicolai T. Wiemers A. Hinrichs B. Amrhein P. . (2021). Pediatric airway endoscopy: recommendations of the society for pediatric pneumology. Respiration 100, 1128–1145. doi: 10.1159/000517125, PMID: 34098560

[B24] Serpa P. H. Deng X. Abdelghany M. Crawford E. Malcolm K. Caldera S. . (2022). Metagenomic prediction of antimicrobial resistance in critically ill patients with lower respiratory tract infections. Genome Med. 14, 74. doi: 10.1186/s13073-022-01072-4, PMID: 35818068 PMC9275031

[B25] Shi T. Chen C. Huang L. Fan H. Lu G. Yang D. . (2020). Risk factors for mortality from severe community-acquired pneumonia in hospitalized children transferred to the pediatric intensive care unit. Pediatr. Neonatol. 61, 577–583. doi: 10.1016/j.pedneo.2020.06.005, PMID: 32651007

[B26] Shi C. L. Han P. Tang P. J. Chen M. M. Ye Z. J. Wu M. Y. . (2020). Clinical metagenomic sequencing for diagnosis of pulmonary tuberculosis. J. Infect. 81, 567–574. doi: 10.1016/j.jinf.2020.08.004, PMID: 32768450

[B27] Smith D. K. Kuckel D. P. Recidoro A. M. (2021). Community-acquired pneumonia in children: rapid evidence review. Am. Fam. Phys. 104, 618–625. 34913645

[B28] Su L. D. Chiu C. Y. Gaston D. Hogan C. A. Miller S. Simon D. W. . (2024). Clinical metagenomic next-generation sequencing for diagnosis of central nervous system infections: advances and challenges. Mol. Diagn. Ther. 28, 513–523. doi: 10.1007/s40291-024-00727-9, PMID: 38992308 PMC11660858

[B29] Sun L. Zhang S. Yang Z. Yang F. Wang Z. Li H. . (2022). Clinical application and influencing factor analysis of metagenomic next-generation sequencing (mNGS) in ICU patients with sepsis. Front. Cell Infect. Microbiol. 12, 905132. doi: 10.3389/fcimb.2022.905132, PMID: 35909965 PMC9326263

[B30] Tao Y. Yan H. Liu Y. Zhang F. Luo L. Zhou Y. . (2022). Diagnostic performance of metagenomic next-generation sequencing in pediatric patients: A retrospective study in a large children’s medical center. Clin. Chem. 68, 1031–1041. doi: 10.1093/clinchem/hvac067, PMID: 35704075

[B31] Thibeault C. Suttorp N. Opitz B. (2021). The microbiota in pneumonia: From protection to predisposition. Sci. Transl. Med. 13 (576), eaba0501. doi: 10.1126/scitranslmed.aba0501, PMID: 33441423

[B32] Walker P. J. Wilkes C. Duke T. Graham H. R . (2022). Can child pneumonia in low-resource settings be treated without antibiotics? A systematic review & meta-analysis. J. Glob. Health 12, 10007. doi: 10.7189/jogh.12.10007, PMID: 36370376 PMC9653171

[B33] Wei Y. Zhang T. Ma Y. Yan J. Zhan J. Zheng J. . (2023). Clinical Evaluation of Metagenomic Next-Generation Sequencing for the detection of pathogens in BALF in severe community acquired pneumonia. Ital. J. Pediatr. 49, 25. doi: 10.1186/s13052-023-01431-w, PMID: 36805803 PMC9938609

[B34] Wu X. Lu W. Wang T. Xiao A. Guo X. Xu Y. . (2023). Optimization strategy for the early timing of bronchoalveolar lavage treatment for children with severe mycoplasma pneumoniae pneumonia. BMC Infect. Dis. 23, 661. doi: 10.1186/s12879-023-08619-9, PMID: 37798699 PMC10557288

[B35] Wu X. Sun T. He H. Xing L. Cheng Z. Geng S. . (2025). Effect of metagenomic next-generation sequencing on clinical outcomes of patients with severe community-acquired pneumonia in the ICU: A multicenter, randomized controlled trial. Chest 167, 362–373. doi: 10.1016/j.chest.2024.07.144, PMID: 39067508

[B36] Yang A. Chen C. Hu Y. Zheng G. Chen P. Xie Z. . (2022). Application of metagenomic next-generation sequencing (mNGS) using bronchoalveolar lavage fluid (BALF) in diagnosing pneumonia of children. Microbiol. Spectr. 10, e0148822. doi: 10.1128/spectrum.01488-22, PMID: 36169415 PMC9603332

[B37] Yao A. Wang J. Xu Q. Shah B. K. Sun K. Hu F. . (2024). Higher diagnostic value of metagenomic next-generation sequencing in acute infection than chronic infection: a multicenter retrospective study. Front. Microbiol. 15, 1295184. doi: 10.3389/fmicb.2024.1295184, PMID: 38351916 PMC10864100

[B38] Zhang P. Liu B. Zhang S. Chang X. Zhang L. Gu D. . (2024). Clinical application of targeted next-generation sequencing in severe pneumonia: a retrospective review. Crit. Care 28, 225. doi: 10.1186/s13054-024-05009-8, PMID: 38978111 PMC11232260

[B39] Zhao X. Bai L. P. Li B. Y. Yue Z. Z. Zhao Y. C. Zhao X. Y. . (2023). Comparison of mNGS and conventional culture in non-organ transplant critically ill patients supported by ECMO: a single-center study. Front. Cell Infect. Microbiol. 13, 1146088. doi: 10.3389/fcimb.2023.1146088, PMID: 37139490 PMC10149872

[B40] Zheng L. Liao W. Liang F. Li K. Li L. Liang H. . (2021). Clinical characteristics and outcomes of severe pneumonia in children under 5 years old with and without adenovirus infection in guangzhou. Front. Pediatr. 9, 599500. doi: 10.3389/fped.2021.599500, PMID: 34869087 PMC8634581

[B41] Zhu Y. Gan M. Ge M. Dong X. Yan G. Zhou Q. . (2023). Diagnostic performance and clinical impact of metagenomic next-generation sequencing for pediatric infectious diseases. J. Clin. Microbiol. 61, e0011523. doi: 10.1128/jcm.00115-23, PMID: 37260394 PMC10281092

